# Supplementary material for: Exploring high mortality rates among people with multiple and complex needs: a qualitative study using peer research methods
Source: BMJ Open. 2021 Jun 3;11(6):e044634. doi: 10.1136/bmjopen-2020-044634 (PMC8183219; doi:10.1136/bmjopen-2020-044634)
Supplement: Author's manuscript [file bmjopen-2020-044634.draft_revisions.pdf]

BMJ Open is committed to open peer review. As part of this commitment we make the peer review history of every article we publish publicly available.

When an article is published we post the peer reviewers' comments and the authors' responses online. We also post the versions of the paper that were used during peer review. These are the versions that the peer review comments apply to.

The versions of the paper that follow are the versions that were submitted during the peer review process. They are not the versions of record or the final published versions. They should not be cited or distributed as the published version of this manuscript.

BMJ Open is an open access journal and the full, final, typeset and author-corrected version of record of the manuscript is available on our site with no access controls, subscription charges or pay-per-view fees (http://bmjopen.bmj.com).

If you have any questions on BMJ Open's open peer review process please email info.bmjopen@bmj.com

BMJ Open

EXPLORING HIGH MORTALITY RATES AMONG PEOPLE WITH MULTIPLE AND COMPLEX NEEDS: A QUALITATIVE STUDY USING PEER RESEARCH METHODS

Journal:	*BMJ Open*
Manuscript ID	bmjopen-2020-044634
Article Type:	Original research
Date Submitted by the Author:	08-Sep-2020

Complete List of Authors:	Perry, Rachel; Newcastle University, Population Health Sciences Institute<br>Adams, Emma; Newcastle University, Population Health Sciences Institute<br>Harland, Jill; Newcastle University, Population Health Sciences Institute<br>Broadbridge, Angela; Fulfilling Lives Newcastle Gateshead<br>Giles, Emma ; Teesside University, School of Health and Life Sciences<br>McGeechan, Grant; Teesside University, Centre for Applied Psychological Science<br>O'Donnell, Amy; Newcastle University, Population Health Sciences Institute<br>Ramsay, Sheena; Newcastle University, Population Health Sciences Institute

Keywords:	PREVENTIVE MEDICINE, PUBLIC HEALTH, QUALITATIVE RESEARCH

SCHOLARONE™
Manuscripts

I, the Submitting Author has the right to grant and does grant on behalf of all authors of the Work (as defined in the below author licence), an exclusive licence and/or a non-exclusive licence for contributions from authors who are: i) UK Crown employees; ii) where BMJ has agreed a CC-BY licence shall apply, and/or iii) in accordance with the terms applicable for US Federal Government officers or employees acting as part of their official duties; on a worldwide, perpetual, irrevocable, royalty-free basis to BMJ Publishing Group Ltd ("BMJ") its licensees and where the relevant Journal is co-owned by BMJ to the co-owners of the Journal, to publish the Work in this journal and any other BMJ products and to exploit all rights, as set out in our licence.

The Submitting Author accepts and understands that any supply made under these terms is made by BMJ to the Submitting Author unless you are acting as an employee on behalf of your employer or a postgraduate student of an affiliated institution which is paying any applicable article publishing charge ("APC") for Open Access articles. Where the Submitting Author wishes to make the Work available on an Open Access basis (and intends to pay the relevant APC), the terms of reuse of such Open Access shall be governed by a Creative Commons licence – details of these licences and which Creative Commons licence will apply to this Work are set out in our licence referred to above.

Other than as permitted in any relevant BMJ Author's Self Archiving Policies, I confirm this Work has not been accepted for publication elsewhere, is not being considered for publication elsewhere and does not duplicate material already published. I confirm all authors consent to publication of this Work and authorise the granting of this licence.

For peer review only

EXPLORING HIGH MORTALITY RATES AMONG PEOPLE WITH MULTIPLE AND COMPLEX NEEDS: A QUALITATIVE STUDY USING PEER RESEARCH METHODS

Rachel Perry, MPH[1]
Emma A. Adams, MSc[1]
Jill Harland, MSc[1]
Angela Broadbridge, MSc[2]
Emma L. Giles, PhD[3]
Grant J. McGeechan, PhD[4]
Amy O'Donnell, PhD[1]
Sheena E. Ramsay, PhD[1]

[1] Population Health Sciences Institute, Newcastle University, Newcastle upon Tyne, United Kingdom
[2] Fulfilling Lives Newcastle Gateshead, Gateshead, United Kingdom
[3] School of Health and Life Sciences, Teesside University, Middlesbrough, United Kingdom
[4] Centre for Applied Psychological Science, Teesside University, Middlesbrough, United Kingdom

Corresponding author:
Emma Audrey Adams
Population Health Sciences Institute
Baddiley-Clark Building
Newcastle University, Newcastle upon Tyne
NE2 4AX, United Kingdom
emma.adams@newcastle.ac.uk
0191 222 7045

Word Count: 3280 words

For peer review only - http://bmjopen.bmj.com/site/about/guidelines.xhtml

ABSTRACT

Objective: To explore the reasons underlying high mortality rates among people with multiple and complex needs and potential preventive opportunities.

Design: Qualitative study using peer research

Setting: North East of England

Participants: Three focus group discussions were held involving: 1) people with lived experience of MCN (n=5); 2) frontline staff from health, social care, and voluntary organisations that support MCN groups (n=7); and 3) managers and commissioners of these organisations (n=9).

Results: Findings from this study provide valuable insights from people with lived experience and staff on the complexity underpinning high mortality rates for individuals experiencing multiple and complex needs. Mental ill-health and substance misuse (often co-occurring dual diagnosis) were highlighted as significant factors underlying premature mortality among multiple and complex needs groups. Potential triggers to identify people at-risk included critical life events (e.g. bereavement, relationship breakdown) and transitions (e.g. release from prison, completion of drug treatment). Early prevention, particularly supporting young people experiencing adverse childhood experiences was also highlighted as a priority.

Conclusion: High mortality in MCN groups may be reduced by addressing dual diagnosis, providing more support at critical life events, and investing in early prevention efforts. Future service delivery should take into consideration the intricate nature of multiple and complex needs and improve service access and navigation.

Keywords: multiple complex needs, mortality, prevention, interventions

ARTICLE SUMMARY

Strengths and limitations of this study

- This study employed focus group discussions with individuals with multiple and complex needs and service providers to understand the complexity underpinning high morality rates for individuals experiencing multiple and complex needs.
- Peer researchers contributed to all stages of this study, including developing the aims, data collection, interpretation, and shaping recommendations.
- Using peer researchers enhanced our access to participants and improved interpretation of data
- The main limitation is that the study only recruited individuals in one region in the North East of England. Views from individuals with MCN and service providers in other areas of England might have led the results to being more generalisable.

For peer review only - http://bmjopen.bmj.com/site/about/guidelines.xhtml

INTRODUCTION

Despite increases in life expectancy in the United Kingdom (UK) throughout the 20th century, improvements in mortality rates have stalled in recent years for most population groups.[1] Since 2010-2011, the gap in life expectancy between people living in the most and least deprived communities has widened, with the largest decreases seen in the 10% most deprived neighbourhoods in the North East of England [2, 3]. This region has also observed high rates of drug-related deaths and suicide in younger adults (under age 50) compared to other parts of the UK.[4]

People facing co-occurring issues of homelessness, substance misuse, offending, and mental ill-health experience particularly poor health outcomes compared to the general population.[5] Multiple complex needs (MCN) has been used to describe this group; other terminology include severe and multiple disadvantage or multiple exclusion homelessness.[5-8] When considered in isolation, each of these issues contributes to higher mortality rates compared to the general population.[9-12] However, there is a growing appreciation of the intersecting nature of these needs and the compounding effect these can have on health outcomes.[5] All-cause mortality rates for people with MCN compared to the UK general population is almost seven times higher for men and almost twelve times higher for women, with major causes of deaths including injury, poisoning, external causes (i.e. accidents, homicide, or suicide), and social exclusion.[13]

Despite such inequality, gaps remain in our understanding of the perspectives of individuals with lived experienced of MCN and those who support them (e.g. health and social care workers) on the factors contributing to high death rates in this population.[14] Involving peer researchers across the continuum of research, from concept development, to data collection, analysis, and dissemination, can help provide valuable access to participants from marginalized populations, as well as offering unique insights on how evidence should be collected and interpreted.[8, 15-17] Peer researchers are individuals who have lived experience of the phenomenon under study and are at a stage where they are ready to support others discuss their own experiences.[8] Although there is increasing recognition of the need to involve peer researchers in health and social care research, there remains limited representation in current literature on mortality and MCN.

The aim of this study was to explore the perspectives of people with lived experience of MCN and professionals who support them in order to understand factors underlying high mortality rates in this population. In doing so, we also sought to obtain rich insights into opportunities for preventing deaths and improving service provision for MCN groups in the future. The study was developed and undertaken with peer researchers.

METHODS

A qualitative study using a peer-research approach was conducted. Peer researchers from Fulfilling Lives Newcastle Gateshead, a project that supports MCN groups, contributed to all stages of this study, including developing the aims, data collection, interpretation, and shaping recommendations. Peer researchers received NVQ-level training in peer research and had lived experience of MCN.

For peer review only - http://bmjopen.bmj.com/site/about/guidelines.xhtml

Data collection

The study sample comprised people with lived experience of MCN, frontline staff, and managers/commissioners of relevant health, social care, and voluntary support services. Peer researchers suggested using focus group discussions (FGDs) to help create a supportive environment for conversations around a sensitive topic. A combination of convenience (using pre-existing networks) and maximum variation sampling techniques were used to recruit participants through email and mailing lists within the North East of England for three homogenous FGDs. Participants were recruited from both operational (frontline) and commissioning/managerial levels to ensure diversity of staff perspectives. Additionally, variation was sought in terms of gender, different components of MCN, and age for participants with lived experience, although all were aged 18 and over. Many peer researchers had relationships with lived experience FGD participants; however, this was a deliberate strategy as it enhanced our access to participants and improved the interpretation of data.

Ethical approval for the study was obtained from Newcastle University Ethics Committee (reference 11064/2018). A participant information sheet was made available to all participants and informed consent was gained prior to data collection.

Three homogenous FGDs were conducted in July 2019 with: individuals with lived experience of MCN (FGD 1, n=5); frontline staff (FGD 2, n=7); and managers/commissioners (FGD 3, n=9) working in health, social care, and voluntary sectors. Representatives from a range of voluntary and statutory organisations participated, including those working in local authority commissioning, mental health, substance misuse and housing and family services. No participants withdrew during the FGDs.

The FGDs were jointly facilitated by an academic and a peer researcher and held at accessible venues in Newcastle upon Tyne. Two semi-structured topic guides (Table 1) were co-produced with peer researchers, one for use with individuals with MCN and one for staff FGDs, with probes used to elicit additional information and detailed responses. All participants were fully briefed on the study (aims, objectives, and dissemination plans) prior to gaining consent. FGDs lasted approximately 90 minutes, and were audio recorded.

Table 1. Topic guides for the focus groups

Topic guide for focus groups with individuals with lived experience of MCN	Topic guide for focus groups with staff working with MCN groups
• Awareness of mortality within their peer group<br>• What factors/life experiences do they think contribute to premature mortality within their peer group.<br>• Any concerns they have about this personally or for others<br>• Do they think anything could have been done to prevent people dying?<br>• Can they describe this?	• Awareness of premature mortality within multiple and complex needs groups<br>• Awareness of risk factors for premature mortality<br>• Current approaches to identify those at risk – perceptions of effectiveness<br>• What would help the services identify/ target those at risk<br>• Current interventions – perceptions of effectiveness

For peer review only - http://bmjopen.bmj.com/site/about/guidelines.xhtml

• What types of help and support would they like to see being developed/ provided?<br>• How would this be best offered?	• Types of interventions/approaches they think should be in place<br>• How could this be taken forward

Data analysis

Audio-recordings were transcribed verbatim and uploaded into NVivo 12 to support data management and analysis. To protect anonymity, particularly given the sensitivity of the topic, any potential identifiers (including participant characteristics such as age, gender, role) were removed. Field notes were used to capture immediate reflections on the FGDs and to aid analysis and interpretation. Peer researchers reviewed transcripts for authenticity. Deductive thematic analysis based on Braun and Clarke's[18] approach was conducted. Initial codes were co-created with peer researchers based on the study aim and objectives, existing literature, and an initial review of the transcripts. Each transcript was coded whilst adaptively developing the code book with any new codes and returning to previous transcripts. The first author conducted the primary coding and a section of each transcript was second coded by two other authors to ensure consistency. Codes were collated into potential themes, which were reviewed amongst the research team and further refined. In addition to peer researchers, the team compromised academic researchers, service providers, and public health registrar trainees with prior research experience. Consensus was reached through discussions with peer researchers and the wider research team on the themes, subthemes, extracts, and recommendations.

Patient and public involvement statement

One of the co-authors (AB) works for Fulfilling Lives Newcastle Gateshead, which is an eight-year learning programm to improve the lives of people with MCN and build a trauma-informed approach with the services that support them. The project design and methodology was developed with a group of Experts by Experience (peer researchers) and fronline staff who work for Fulfilling Lives Newcastle Gateshead. As previously mentioned, peer researchers contributed to all stages of the research. The methodology selected for data collection (FGDs) was choosen based on suggestions from Peer Researchers.

RESULTS

Findings were relatively homogenous across all three FGDs. Findings are presented according to the two main study objectives and associated themes: *understanding factors underlying premature mortality* amongst MCN groups and *opportunities for interventions to prevent mortality.*

Understanding factors underlying premature mortality in MCN groups

Burden of mental ill-health and substance misuse issues

For peer review only - http://bmjopen.bmj.com/site/about/guidelines.xhtml

The severe burden of mental ill-health in people with MCN was a recurrent concern and felt to make a substantial contribution to premature mortality, particularly in relation to mental illness alongside substance misuse (dual diagnosis).

> *"Most of the people I know that's died, their mental health has just been shot to bits, it's all about the drugs. They're taking the drugs because of mental health, is that bad? I'd say more the mental health killed them…the drugs just done that job"*
>
> - *Individual with lived experience of MCN*

There was a perception that people with MCN experienced challenges when trying to access services and support for co-existing mental ill health and substance misuse. Services were set up to deal with either mental health issues, or addiction issues, meaning those with a dual diagnosis failed to receive appropriate support.

> *"I just find that people who have got mental-health issues and also have addiction problems fall through the gaps, time and time again"*
>
> - *Frontline staff*

Lack of hope, stigma and health-seeking behaviour

Participants with MCN highlighted that experiences of loss were so common that it led to individuals becoming desensitized to deaths and reaching a point of acceptance. Outcomes for individuals experiencing MCN were so often negative this led to a lack of hope that it could ever be any different.

> *"You don't see another way…it's just doom and gloom and like you say this one's dead, this one's in prison, there's nothing ever…it's like being in the sort of devil's dungeon, to be honest"*
>
> - *Individual with lived experience of MCN*

Stigma was highlighted as playing a large role in health-seeking behaviour. Individuals with lived experience expressed frustration around the stigmatizing nature of the words professionals used to describe different facets of MCN.

> *" I think that word as well, like, junky really boils my blood, heroin addict is much better. Just picking up the junky, junky, junky, that's all we get."*
>
> - *Individual with lived experience of MCN*

This fear of stigma along with not being given sufficient time for staff to understand their unique experiences led to individuals avoiding services. FGDs with staff reiterated this perception that many young people they worked with felt intimidated when going to see their General Practitioner and instead chose to self-medicate with illicit substances.

> *"… this doctor at the time of [the] appointment isn't going to be able to comprehend even a tiny touch of what your life is."*

For peer review only - http://bmjopen.bmj.com/site/about/guidelines.xhtml

- Individual with lived experience of MCN

"We talked to the young people who have got mental health issues and they're kind of like, "Oh no, I don't want to talk to anyone, I don't want to tell them I've got a problem. I'd rather just smoke some weed or take some grass and I'll be okay."

- Manager/Commissioner

Poor navigation and limited services

Across all three FGDs, participants expressed problems with navigating service pathways, limited services and having to engage with traditional models of care. Individuals with MCN felt as though they were faced with a series of barriers to gaining appropriate support, which increased the vulnerability of MCN groups to premature mortality.

"I'm facing this maze full of doors and every time I open a door, there's another door, sets of doors. There's no coherent structure within the system that says, "Here's a person who is asking for help, who's engaging with everything that we're giving, can we please pull this together so we can actually provide the help that this person needs."

- Individual with lived experience of MCN

There was a perception that recent reductions in available support services meant that the support was often only provided in extreme circumstances, such as when an individual had reached a crisis point.

"it's often such a desperate situation that we're having ridiculous conversations that we want someone to be sectioned or we want someone to go to prison just so they're in some kind of contained environment where we feel we can try and manage some of the risks"

- Manager/Commissioner

Opportunities for interventions to prevent morality in MCN groups

Intervention timing

Critical life events (e.g. childhood adversity and bereavement) and transitions (e.g. release from prison) were highlighted as moments of key vulnerability, as well as potential opportunities for intervention. Participants felt that many of the issues encountered by people experiencing MCN were rooted in early childhood. Therefore, improved support for young people who experience adverse childhood experiences could prevent the development and exacerbation of long-term needs.

"Preventive measures early on may stop the numbers of people coming through with multiple and complex needs. So it's the preventive, it's the community centres, it's the youth centres, it's those things where the learning happens."

- Manager/Commissioner

For peer review only - http://bmjopen.bmj.com/site/about/guidelines.xhtml

Participants in all focus groups agreed that windows of opportunity (time points of places where services could be provided) are often brief and difficult to take advantage of.

> *"There's often an inability to exploit windows of opportunity where…support workers will try and get all their ducks in a row. So the mental health stuff, the mental health treatment, housing, benefits, all of that sort of stuff, it's rare that you're going to manage to get all of that sorted in the two hours of window opportunity you've got. Then the ship sails sometimes and you don't know whether that's going to come back again or when it's going to come back again."*
>
> - *Manager/commissioner*

Intervention approaches

Participants provided suggestions for how interventions could be improved in future. The need for a holistic, person-centred approach was highlighted, acknowledging that a "one-size-fits-all" approach was unlikely to cover the complexity of need or empower individuals to engage.

> *"I think there needs to be focus on it being really a person-centred approach and say, "This isn't working for me at the moment and that's how I would like things to be," and giving them that sense of responsibility"*
>
> - *Frontline staff*

Building a sense of community for people living with MCN was a common suggestion aimed at reducing social exclusion. The value of peer support communities in particular was highlighted.

> *"There's a connection [as a peer supporter]. Immediately there's a connection but through that connection you feel like you've gotten in and you feel like what you say has a better chance of making a difference to that person"*
>
> - *Individual with lived experience of MCN*

The need for better collaboration across the multiple agencies involved in supporting people living with MCN was also highlighted. In particular, improved communication between services, and the opportunity to share learning across sectors were suggested as ways to reduce missed opportunities for prevention.

> *"We exist in a competitive tendering landscape and we need to leave that aside and come together and share good practice and learn from what's happening across the world"*
>
> - *Manager/Commissioner*

DISCUSSION

This study used FGDs to gather insights from individuals with lived experience, frontline workers and those responsible for managing and commission services on factors underlying high mortality rates in MCN populations and opportunities for potential interventions. Across discussions, we found that issues relating to the burden of mental ill-health and substance misuse (dual diagnosis), combined with experiencing stigma and exclusion when accessing services,

For peer review only

were felt to contribute to adverse mortality outcomes in this group. Participants highlighted key life events (e.g. childhood adversity) and transitions (e.g. release from prison), as potential opportunities for targeted intervention to better support this population in the future.

Epidemiological data confirms the contribution of mental ill-health and co-occurring substance misuse to the high mortality rates in people experiencing MCN.[12, 19] Homeless individuals have greater prevalence of alcohol use disorders than the general population,[20] and self-reported data suggest that mental health problems are as high as 92% among individuals with MCN.[5] This study provides valuable qualitative insights from both people living with MCN and those delivering and commissioning services as to how these issues combine to affect health outcomes. As others have reported, we found that the reality of many individuals with MCN is a journey dominated by the challenge of navigating a siloed, highly fragmented system, that is ill-equipped to meet their needs.[21] Not unique to this study, are the experiences of many people with MCN finding the co-occurring nature of issues leads to an inability to access services or falling through system "cracks."[22] However, we also highlight the stigma and discrimination experienced by people with MCN as they attempt to access professional services designed to provide help, which represents a further barrier to support, as well as contributing to a deep sense of hopelessness amongst this population.

A recent mapping exercise of MCN across England found the current system of support across public and voluntary services was struggling to meet present demands and deliver positive outcomes for individuals with MCN.[5] This is consistent with experiences shared throughout our study. Participants in the focus groups pointed to issues with the present system and identified that early prevention and targeting interventions at "critical life events" (i.e. transitions in service provision or at experiences of heightened adversity) could be important in reducing deaths in MCN groups. There was a particular emphasis on creating interventions targeting youth to address concerns earlier on in the prevention spectrum. Evidence highlighting the link between experiences of childhood adversity and later life MCN reiterate the need to create preventative interventions surrounding this time period.[5, 7, 23] Additionally, our findings suggest that future provision should focus on interventions that are developed collaboratively across sectors [5, 24] targeting critical life events [7, 23] using person-centred and trauma-informed practices,[24, 25] and peer support.[26]

The novelty of this study is it has shown the value of listening to individuals with experience of MCN. It enabled an exploration of an issue that directly affects their community and supported understanding of some of the underlying issues, as well as identifying some avenues for possible preventive interventions. This enrichment of the research aligns with existing literature exploring peer research use for vulnerable populations or sensitive topics.[8, 15-17] Furthermore, the insights specific to opportunities for service provision take into consideration the lived experience of the target population, which can lead to more equitable service delivery and engagement.[16]

Limitations

As our study was located in the North East of England, our findings may not be generalisable to other regions. Response bias is a possibility as individuals with stronger views might have been more likely to volunteer participate in a study. However, since focus groups were used, we feel

For peer review only - http://bmjopen.bmj.com/site/about/guidelines.xhtml

that the potential effects were reduced as the groups allowed for a range of views to be considered. There were limitations in the representativeness of individuals with MCN; a recruitment challenge experienced in previous studies.[16] Nonetheless, by engaging peer researchers throughout the research process, we feel this potential limitation was reduced. There is also the risk that participants might have felt more comfortable speaking to an outsider rather than a peer about certain issues.[17] However, as the study focused less on the individual and more on the overarching issues related to mortality rates, this was likely minimal.

CONCLUSIONS

Mortality rates among individuals experiencing MCN in the UK continue to rise due to an interconnected web of disadvantage and exclusion. Findings from this study provide valuable insights from people with lived experience and staff supporting MCN groups on the complexity underpinning this trend. Reducing mortality rates among MCN groups requires addressing issues related to dual diagnosis, providing support around critical life events, and investing in early prevention. The intersecting nature of MCN should be factored into the design of future interventions to address the challenges related to service navigation and availability. The need to address the underlying causes of MCN and develop effective and sustainable interventions to address high mortality rates has become even more important in light of the COVID-19 pandemic, which has exacerbated issues around service access, health inequalities, and social isolation.[27]

For peer review only

Acknowledgements:

We would like to acknowledge the various Peer Researchers from Fulfilling Lives Newcastle Gateshead who contributed to all stages of this study including developing the aim, methods, data collection, and interpretation. As well, we appreciate the time and effort of all participants who offered their views on and experiences of this challenging and emotive topic.

Funding statement:

This work was funded by a small seed grant from Public Health England as part of the Research Hub Initiative. EAA, ELG, GJM, AOD, and SR, are members of Fuse, the Centre for Translational Research in Public Health (www.fuse.ac.uk). Fuse is a UK Clinical Research Collaboration (UKCRC) Public Health Research Centre of Excellence. Funding for Fuse from the British Heart Foundation, Cancer Research UK, National Institute of Health Research, Economic and Social Research Council, Medical Research Council, Health and Social Care Research and Development Office, Northern Ireland, National Institute for Social Care and Health Research (Welsh Assembly Government) and the Wellcome Trust, under the auspices of the UKCRC, is gratefully acknowledged. EAA is supported by the National Institute for Health Research (NIHR) School for Public Health Research (SPHR) Pre-doctoral Fellowship, Grant Reference Number PD-SPH-2015. SR and AOD are members of the National Institute for Health Research (NIHR) Applied Research Collaboration (ARC) North East & North Cumbria Inequality Theme. The views expressed are those of the author(s) and not necessarily those of the NIHR, Department of Health and Social Care, Public Health England, or any of the other funding or organizational bodies.

Declaration of interests:

All authors declare no competing interests.

Data sharing:

Due to the sensitivity of the topic and the numbers of participants, we are not able to share FGD transcripts. Summaries are available from the corresponding author on request.

Author contributions:

Conceptualization: JH, SR, AOD, AB, GJM, ELG and RP
Data acquisition: JH, AB, and SR
Analysis: RP, AB, ELG, GJM, and EAA
Interpretation: All authors contributed to the interpretation of findings.
Writing: All authors contributed to the writing of the paper.
All authors have read and approved the final version to be submitted for consideration for publication.

For peer review only

References:

1. Marshall L, Finch D, Cairncross L, et al. Mortality and life expectancy trends in the UK: Stalling progress. London: The Health Foundation, 2019. Available: https://reader.health.org.uk/mortality-trends-in-the-uk [Accessed Aug 2020].

2. Marmot M, Allen J, Boyce T, et al. Health equity in England: The marmot review 10 years on. London; 2020. Available: https://www.health.org.uk/publications/reports/the-marmot-review-10-years-on [Accessed Aug 2020].

3. Marmot M. Health equity in England: The marmot review 10 years on. *BMJ* 2020;368:m693. doi: 10.1136/bmj.m693.

4. Office for National Statistics. Drug-related deaths by local authority: England and wales. 2019. Available: www.ons.gov.uk/peoplepopulationandcommunity/birthsdeathsandmarriages/deaths/datasets/drugmisusedeathsbylocalauthority [Accessed Aug 2020].

5. Bramley G, Fitzpatrick S, Edwards J, et al. Hard edges: Mapping severe and multiple disadvantage (England). London: Lankelly Chase Foundation 2015. Available: https://lankellychase.org.uk/wp-content/uploads/2015/07/Hard-Edges-Mapping-SMD-2015.pdf [Accessed Aug 2020].

6. Rosengard A, Laing I, Ridley J, et al. A literature review on multiple and complex needs. Edinburgh: Scottish Executive Social Research 2007. Available: https://www.webarchive.org.uk/wayback/archive/20170112055441/http://www.gov.scot/Publications/2007/01/18133419/15 [Accessed Aug 2020].

7. Fitzpatrick S, Bramley G, Johnsen S. Pathways into multiple exclusion homelessness in seven UK cities. *Urban Stud*2012;50(1):148-68. doi:10.1177/0042098012452329.

8. Terry L, Cardwell V. Refreshing perspectives: Exploring the application of peer research with populations facing severe and multiple disadvantage. London: Revolving Doors Agency & Lankelly Chase 2016. Available: http://www.revolving-doors.org.uk/file/1849/download?token=Yi0tjhmo [Accessed Aug 2020].

9. John A, McGregor J, Jones I, et al. Premature mortality among people with severe mental illness — new evidence from linked primary care data. *Schizophr Res*2018;199:154-62. doi:10.1016/j.schres.2018.04.009.

10. Arendt M, Munk-Jørgensen P, Sher L, et al. Mortality among individuals with cannabis, cocaine, amphetamine, mdma, and opioid use disorders: A nationwide follow-up study of Danish substance users in treatment. *Drug Alcohol Depend*2011;114(2):134-39. doi:10.1016/j.drugalcdep.2010.09.013.

11. Graham L, Fischbacher CM, Stockton D, et al. Understanding extreme mortality among prisoners: A national cohort study in Scotland using data linkage. *Eur J Public Health*2015;25(5):879-85. doi:10.1093/eurpub/cku25.

For peer review only - http://bmjopen.bmj.com/site/about/guidelines.xhtml

12. Thomas B. Homelessness kills: An analysis of the mortality of homeless people in early twenty-first century England. London: Crisis 2012. Available: https://www.crisis.org.uk/media/236798/crisis_homelessness_kills2012.pdf [Accessed Aug 2020].

13. Aldridge RW, Story A, Hwang SW, et al. Morbidity and mortality in homeless individuals, prisoners, sex workers, and individuals with substance use disorders in high-income countries: A systematic review and meta-analysis. *Lancet*2018;391(10117):241-50. doi:10.1016/S0140-6736(17)31869-X.

14. Pope C, Mays N. Qualitative research in health care. 4 ed. GB: Wiley-Blackwell 2019.

15. Woodall J, Cross R, Kinsella K, et al. Using peer research processes to understand strategies to support those with severe, multiple and complex health needs. *Health Education Journal*2019;78(2):176-88. doi:10.1177/0017896918796044.

16. Bonevski B, Randell M, Paul C, et al. Reaching the hard-to-reach: A systematic review of strategies for improving health and medical research with socially disadvantaged groups. *BMC Med Res Methodol*2014;14(1):42-42. doi:10.1186/1471-2288-14-42.

17. Elliott E, Watson AJ, Harries U. Harnessing expertise: Involving peer interviewers in qualitative research with hard-to-reach populations. *Health Expect*2002;5(2):172-78. doi:10.1046/j.1369-6513.2002.00158.x.

18. Braun V, Clarke V. Using thematic analysis in psychology. *Qual Res Psychol*2006;3(2):77-101. doi:10.1191/1478088706qp063oa.

19. Hjemsæter AJ, Bramness JG, Drake R, et al. Mortality, cause of death and risk factors in patients with alcohol use disorder alone or poly-substance use disorders: A 19-year prospective cohort study. *BMC Psychiatry*2019;19(1):101. doi:10.1186/s12888-019-2077-8.

20. Fazel S, Khosla V, Doll H, et al. The prevalence of mental disorders among the homeless in western countries: Systematic review and meta-regression analysis. *PLoS Med*2008;5(12):e225. doi:10.1371/journal.pmed.0050225.

21. Terry L, Cardwell V. Understanding the whole person. London: Revolving Doors Agency & Lankelly Chase 2015. Available: https://lankellychase.org.uk/wp-content/uploads/2015/12/Understanding-the-whole-person-Part-One.pdf [Accessed Aug 2020].

22. St. Mungo's. Dying on the streets: The case for moving quickly to end rough sleeping. 2018. Available: https://www.mungos.org/app/uploads/2018/06/Dying-on-the-Streets-Report.pdf [Accessed Aug 2020].

23. McDonagh T. Tackling homelessness and exclusion: Understanding complex lives. York: Joseph Rowntree Foundation 2011. Available: https://www.jrf.org.uk/sites/default/files/jrf/migrated/files/homelessness-exclusion-services-summary.pdf [Accessed Aug 2020].

For peer review only - http://bmjopen.bmj.com/site/about/guidelines.xhtml

24. Department for Communities and Local Government. Making every contact count: A joint approach to preventing homelessness. London: DCLG 2012. Available: https://assets.publishing.service.gov.uk/government/uploads/system/uploads/attachment_data/file/7597/2200459.pdf [Accessed Aug 2020].

25. Communities and Local Government. Mental health good practice guide. Meeting the psychological and emotional needs of homeless people. London: CLG and National Mental Health Development Unit 2010. Available: https://lx.iriss.org.uk/sites/default/files/resources/meeting-the-psychological-and-emotional-needs-of-people-who-are-homeless.pdf [Accessed Aug 2020].

26. Miler JA, Carver H, Foster R, et al. Provision of peer support at the intersection of homelessness and problem substance use services: A systematic 'state of the art' review. *BMC Public Health*2020;20(1):641. doi:10.1186/s12889-020-8407-4.

27. Douglas M, Katikireddi SV, Taulbut M, et al. Mitigating the wider health effects of covid-19 pandemic response. *BMJ*2020;369:m1557. doi:10.1136/bmj.m1557.

For peer review only

Manuscript: Exploring high mortality rates among people with multiple and complex needs: a qualitative study using peer research methods

Consolidated criteria for reporting qualitative studies (COREQ): 32-item checklist

No. Item	Guide questions/description	Reported on Page #
**Domain 1: Research team and reflexivity**		
Personal Characteristics		
1. Interviewer/facilitator	Which author/s conducted the inter view or focus group?	4
2. Credentials	What were the researcher's credentials? E.g. PhD, MD	1<br>3-5
3. Occupation	What was their occupation at the time of the study?	2,5
4. Gender	Was the researcher male or female?	Not reported
5. Experience and training	What experience or training did the researcher have?	2,5
Relationship with participants		
6. Relationship established	Was a relationship established prior to study commencement?	4
7. Participant knowledge of the interviewer	What did the participants know about the researcher? e.g. personal goals, reasons for doing the research	4
8. Interviewer characteristics	What characteristics were reported about the inter viewer/facilitator? e.g. Bias, assumptions, reasons and interests in the research topic	4
**Domain 2: study design**		
Theoretical framework		
9. Methodological orientation and Theory	What methodological orientation was stated to underpin the study? e.g. grounded theory, discourse analysis, ethnography, phenomenology, content analysis	4
Participant selection		

For peer review only

10. Sampling	How were participants selected? e.g. purposive, convenience, consecutive, snowball	4
11. Method of approach	How were participants approached? e.g. face-to-face, telephone, mail, email	4
12. Sample size	How many participants were in the study?	4
13. Non-participation	How many people refused to participate or dropped out? Reasons?	4
Setting		
14. Setting of data collection	Where was the data collected? e.g. home, clinic, workplace	4
15. Presence of non-participants	Was anyone else present besides the participants and researchers?	4
16. Description of sample	What are the important characteristics of the sample? e.g. demographic data, date	4
Data collection		
17. Interview guide	Were questions, prompts, guides provided by the authors? Was it pilot tested?	4
18. Repeat interviews	Were repeat interviews carried out? If yes, how many?	No
19. Audio/visual recording	Did the research use audio or visual recording to collect the data?	4
20. Field notes	Were field notes made during and/or after the interview or focus group?	4
21. Duration	What was the duration of the interviews or focus group?	4
22. Data saturation	Was data saturation discussed?	5
23. Transcripts returned	Were transcripts returned to participants for comment and/or correction?	4
**Domain 3: analysis and findings**		
Data analysis		
24. Number of data coders	How many data coders coded the data?	4-5
25. Description of the coding tree	Did authors provide a description of the coding tree?	4-5
26. Derivation of themes	Were themes identified in advance or derived from the data?	4-5
27. Software	What software, if applicable, was used to manage the data?	4
28. Participant checking	Did participants provide feedback on the	4

For peer review only

	findings?	
Reporting		
29. Quotations presented	Were participant quotations presented to illustrate the themes/findings? Was each quotation identified? e.g. participant number	5-8
30. Data and findings consistent	Was there consistency between the data presented and the findings?	5-8
31. Clarity of major themes	Were major themes clearly presented in the findings?	5-8
32. Clarity of minor themes	Is there a description of diverse cases or discussion of minor themes?	5-8

For peer review only

EXPLORING HIGH MORTALITY RATES AMONG PEOPLE WITH MULTIPLE AND COMPLEX NEEDS: A QUALITATIVE STUDY USING PEER RESEARCH METHODS

Journal:	*BMJ Open*
Manuscript ID	bmjopen-2020-044634.R1
Article Type:	Original research
Date Submitted by the Author:	26-Jan-2021
Complete List of Authors:	Perry, Rachel; Newcastle University, Population Health Sciences Institute<br>Adams, Emma; Newcastle University, Population Health Sciences Institute<br>Harland, Jill; Newcastle University, Population Health Sciences Institute<br>Broadbridge, Angela; Fulfilling Lives Newcastle Gateshead<br>Giles, Emma ; Teesside University, School of Health and Life Sciences<br>McGeechan, Grant; Teesside University, Centre for Applied Psychological Science<br>O'Donnell, Amy; Newcastle University, Population Health Sciences Institute<br>Ramsay, Sheena; Newcastle University, Population Health Sciences Institute
**Primary Subject Heading**:	Public health
Secondary Subject Heading:	Qualitative research
Keywords:	PREVENTIVE MEDICINE, PUBLIC HEALTH, QUALITATIVE RESEARCH

SCHOLARONE™ Manuscripts

BMJ

I, the Submitting Author has the right to grant and does grant on behalf of all authors of the Work (as defined in the below author licence), an exclusive licence and/or a non-exclusive licence for contributions from authors who are: i) UK Crown employees; ii) where BMJ has agreed a CC-BY licence shall apply, and/or iii) in accordance with the terms applicable for US Federal Government officers or employees acting as part of their official duties; on a worldwide, perpetual, irrevocable, royalty-free basis to BMJ Publishing Group Ltd ("BMJ") its licensees and where the relevant Journal is co-owned by BMJ to the co-owners of the Journal, to publish the Work in this journal and any other BMJ products and to exploit all rights, as set out in our licence.

The Submitting Author accepts and understands that any supply made under these terms is made by BMJ to the Submitting Author unless you are acting as an employee on behalf of your employer or a postgraduate student of an affiliated institution which is paying any applicable article publishing charge ("APC") for Open Access articles. Where the Submitting Author wishes to make the Work available on an Open Access basis (and intends to pay the relevant APC), the terms of reuse of such Open Access shall be governed by a Creative Commons licence – details of these licences and which Creative Commons licence will apply to this Work are set out in our licence referred to above.

Other than as permitted in any relevant BMJ Author's Self Archiving Policies, I confirm this Work has not been accepted for publication elsewhere, is not being considered for publication elsewhere and does not duplicate material already published. I confirm all authors consent to publication of this Work and authorise the granting of this licence.

For peer review only

EXPLORING HIGH MORTALITY RATES AMONG PEOPLE WITH MULTIPLE AND COMPLEX NEEDS: A QUALITATIVE STUDY USING PEER RESEARCH METHODS

Rachel Perry, MPH[1]
Emma A. Adams, MSc[1]
Jill Harland, MSc[1]
Angela Broadbridge, MSc[2]
Emma L. Giles, PhD[3]
Grant J. McGeechan, PhD[4]
Amy O'Donnell, PhD[1]
Sheena E. Ramsay, PhD[1]

[1] Population Health Sciences Institute, Newcastle University, Newcastle upon Tyne, United Kingdom
[2] Fulfilling Lives Newcastle Gateshead, Gateshead, United Kingdom
[3] School of Health and Life Sciences, Teesside University, Middlesbrough, United Kingdom
[4] Centre for Applied Psychological Science, Teesside University, Middlesbrough, United Kingdom

Corresponding author:
Emma Audrey Adams
Population Health Sciences Institute
Baddiley-Clark Building
Newcastle University, Newcastle upon Tyne
NE2 4AX, United Kingdom
emma.adams@newcastle.ac.uk
0191 222 7045

Word Count: 3336 words

For peer review only

For peer review only - http://bmjopen.bmj.com/site/about/guidelines.xhtml

ABSTRACT

Objective: To explore the perceived reasons underlying high mortality rates among people with multiple and complex needs.

Design: Qualitative study using peer research

Setting: North East of England

Participants: Three focus group discussions were held involving: 1) people with lived experience of multiple and complex needs (n=5); 2) frontline staff from health, social care, and voluntary organisations that support multiple and complex needs groups (n=7); and 3) managers and commissioners of these organisations (n=9).

Results: Findings from this study provide valuable perspectives of people with MCN and those that provide them with support on what may be perceived factors underlying premature mortality. Mental ill-health and substance misuse (often co-occurring dual diagnosis) were perceived as influencing premature mortality among multiple and complex needs groups. Perceptions of opportunities for identify people at-risk included critical life events (e.g. bereavement, relationship breakdown) and transitions (e.g. release from prison, completion of drug treatment). Early prevention, particularly supporting young people experiencing adverse childhood experiences was also highlighted as a priority.

Conclusion: High mortality in multiple and complex needs groups may be reduced by addressing dual diagnosis, providing more support at critical life events, and investing in early prevention efforts. Future interventions could take into consideration the intricate nature of multiple and complex needs and improve service access and navigation.

Keywords: multiple complex needs, mortality, prevention, interventions

ARTICLE SUMMARY

Strengths and limitations of this study

- This study employed focus group discussions with individuals with multiple and complex needs and service providers to explore factors perceived to contribute to high premature mortality rates for individuals experiencing multiple and complex needs.
- Peer researchers contributed to all stages of this study, including developing the aims, data collection, interpretation, and shaping recommendations.
- Using peer researchers enhanced our access to participants and improved interpretation of data
- The main limitation is that the study only recruited individuals in one region in the North East of England. Views from individuals with multiple and complex needs and service providers in other areas of England might have led the results to being more generalizable; however, this study was focused on a local level.

For peer review only

INTRODUCTION

Despite increases in life expectancy in the United Kingdom (UK) throughout the 20th century, improvements in mortality rates have stalled in recent years for most population groups.(1) Since 2010-2011, the gap in life expectancy between people living in the most and least deprived communities has widened, with the largest decreases seen in the 10% most deprived neighbourhoods in the North East of England. (2, 3) This region has also observed high rates of drug-related deaths and suicide in younger adults (under age 50) compared to other parts of the UK.(4)

People facing co-occurring issues of homelessness, substance misuse, offending, and mental ill-health experience particularly poor health outcomes compared to the general population.(5) Multiple complex needs (MCN) has been used to describe this group; other terminology include severe and multiple disadvantage or multiple exclusion homelessness.(5-8) Homeless individuals have greater prevalence of alcohol use disorders than the general population,(9) and self-reported data suggest that mental health problems are as high as 92% among individuals with MCN.(5) When considered in isolation, homelessness, substance misuse, mental ill-health, and offending, each contribute to higher mortality rates compared to the general population.(10-13) However, there is a growing appreciation of the intersecting nature of these needs and the compounding effect these can have on health outcomes.(5) The connection between homelessness and premature mortality has been highlighted through a number of quantitative studies.(4, 10, 13-17) Epidemiological data suggests that the contribution of mental ill-health and co-occurring substance misuse is high for mortality rates among people experiencing MCN.(13, 17) All-cause mortality rates for people with MCN compared to the UK general population is almost seven times higher for men and almost twelve times higher for women, with major causes of deaths including injury, poisoning, external causes (i.e. accidents, homicide, or suicide), and social exclusion.(14) However, most of the research into mortality within this population has been epidemiological in nature and there is a gap in qualitative research.

Despite the breath of quantitative and epidemiological data on MCN and mortality, there are very limited perspectives of individuals with lived experienced of MCN and those who support them (e.g. health and social care workers) on the high death rates in this population. A recent mapping exercise of MCN across England found the current system of support across public and voluntary services was struggling to meet present demands and deliver positive outcomes for individuals with MCN.(5) This is increasingly problematic when the system is fragmented and siloed causing added barriers to much needed support. (18, 19) With a system that is not designed to meet the needs of MCN populations, there is a pressing need to conduct research to explore the perspectives of both service users and providers to develop a more comprehensive picture of current challenges and potential opportunities for improvements.

Involving peer researchers across the continuum of research, from concept development, to data collection, analysis, and dissemination, can help provide valuable access to participants from marginalized populations, as well as offering unique insights on how evidence should be collected and interpreted.(8, 20-22) Peer researchers are individuals who have lived experience of the phenomenon under study and are at a stage where they are ready to support others discuss their own experiences.(8) Conducting research *on* MCN populations can be extremely

For peer review only - http://bmjopen.bmj.com/site/about/guidelines.xhtml

challenging; however, conducting studies *with* individuals with lived experience of MCN has been found to add immense value through enhancing recruitment efforts and the collective understanding of the experiences.(23-25) Although there is increasing recognition of the need to involve peer researchers in health and social care research, there remains limited representation in current literature on mortality and MCN.

The aim of this study was to explore the perspectives of people with lived experience of MCN and professionals who support them in order to understand factors perceived to contribute to high mortality rates in this population. This study uses peer research methods to obtain perspectives on premature mortality and rich insights into opportunities for preventing deaths and improving service provision for MCN groups in the future.

For peer review only

METHODS

A qualitative study using a peer-research approach was conducted. Peer researchers from Fulfilling Lives Newcastle Gateshead, a project that supports MCN groups, contributed to all stages of this study, including developing the aims, data collection, interpretation, and shaping recommendations. Peer researchers received NVQ (National Vocational Qualification)-level training in peer research and had lived experience of MCN. Peer researchers identified the study topic as an area that was personally impacting them and required attention. Additionally, peer researchers determined the data collection approach (FGDs), co-developed the topic guide, assisted facilitating focus groups, contextualised the findings, selected quotes, presented findings at a dissemination event, and co-authored a blog on the topic.

Data collection

The study sample comprised people with lived experience of MCN, frontline staff, and managers/commissioners of relevant health, social care, and voluntary support services. Peer researchers decided to use FGDs to help create a supportive environment for conversations around a sensitive topic. A combination of convenience (using pre-existing networks) and maximum variation sampling techniques were used to recruit participants through email and mailing lists within the North East of England for three homogenous FGDs. Participants were recruited from both operational (frontline) and commissioning/managerial levels to ensure diversity of staff perspectives. Additionally, variation was sought in terms of gender, different components of MCN, and age for participants with lived experience, although all were aged 18 and over. Many peer researchers had relationships with lived experience FGD participants; however, this was a deliberate strategy as it enhanced our access to participants and improved the interpretation of data.

Ethical approval for the study was obtained from Newcastle University Ethics Committee (reference 11064/2018). A participant information sheet was made available to all participants and informed consent was gained prior to data collection.

For peer review only - http://bmjopen.bmj.com/site/about/guidelines.xhtml

Three homogenous FGDs were conducted in June/July 2019 with: individuals with lived experience of MCN (FGD 1, n=5); frontline staff (FGD 2, n=7); and managers/commissioners (FGD 3, n=9) working in health, social care, and voluntary sectors. Representatives from a range of voluntary and statutory organisations participated, including those working in local authority commissioning, mental health, substance misuse and housing and family services. Available participant characteristics are reported in Table 1. No participants withdrew during the FGDs.

Table 1. Participant characteristics for the three focus groups

Individual with lived experience FGD	Frontline staff FGD	Manager/Commissioner FGD
5 participants<br>• 2 females<br>• 3 males	7 participants<br>• 6 females<br>• 1 male<br><br>6 different organisations representing service provision for the following:<br>• 2 Mental health<br>• 1 Housing<br>• 1 Homelessness<br>• 1 MCN<br>• 1 Drug and alcohol	9 participants<br>• 6 females<br>• 3 males<br><br>8 different organisations representing service provision for the following:<br>• 2 Housing<br>• 2 Mental health<br>• 2 MCN<br>• 1 Local council/government<br>• 1 Drug and alcohol

The FGDs were jointly facilitated by an academic and a peer researcher and held at accessible venues in Newcastle upon Tyne. Two semi-structured topic guides (Table 2) were co-produced with peer researchers, one for use with individuals with MCN and one for staff FGDs, with probes used to elicit additional information and detailed responses. All participants were fully briefed on the study (aims, objectives, and dissemination plans) prior to gaining consent. FGDs lasted approximately 90 minutes, and were audio recorded.

Table 2. Topic guides for the focus groups

Topic guide for focus groups with individuals with lived experience of MCN	Topic guide for focus groups with staff working with MCN groups
• Awareness of mortality within their peer group<br>• What factors/life experiences do they think contribute to premature mortality within their peer group.<br>• Any concerns they have about this personally or for others<br>• Do they think anything could have been done to prevent people dying?<br>• Can they describe this?<br>• What types of help and support would they like to see being developed/ provided?<br>• How would this be best offered?	• Awareness of premature mortality within multiple and complex needs groups<br>• Awareness of risk factors for premature mortality<br>• Current approaches to identify those at risk – perceptions of effectiveness<br>• What would help the services identify/ target those at risk<br>• Current interventions – perceptions of effectiveness<br>• Types of interventions/approaches they think should be in place<br>• How could this be taken forward

For peer review only - http://bmjopen.bmj.com/site/about/guidelines.xhtml

Data analysis

Audio-recordings were transcribed verbatim and uploaded into NVivo 12 to support data management and analysis. To protect anonymity, particularly given the sensitivity of the topic, any potential identifiers (including participant characteristics such as age, gender, role) were removed. Field notes were used to capture immediate reflections on the FGDs and to aid analysis and interpretation. Peer researchers reviewed transcripts for authenticity. Deductive thematic analysis based on Braun and Clarke's(26) approach was conducted. Initial codes were co-created with peer researchers based on the study aim and objectives, existing literature, and an initial review of the transcripts. Each transcript was coded whilst adaptively developing the code book with any new codes and returning to previous transcripts. The first author conducted the primary coding and a section of each transcript was second coded by two other authors to ensure consistency. Codes were collated into potential themes, which were reviewed amongst the research team and further refined. In addition to peer researchers, the team compromised academic researchers, service providers, and public health registrar trainees with prior research experience. Consensus was reached through discussions with peer researchers and the wider research team on the themes, subthemes, extracts, and recommendations.

Patient and public involvement statement

One of the co-authors (AB) works for Fulfilling Lives Newcastle Gateshead, which is an eight-year learning programme to improve the lives of people with MCN and build a trauma-informed approach with the services that support them. The project design and methodology were developed with a group of Experts by Experience (peer researchers) and frontline staff who work for Fulfilling Lives Newcastle Gateshead. As previously mentioned, peer researchers contributed to all stages of the research. The methodology selected for data collection (FGDs) was chosen based on suggestions from Peer Researchers.

RESULTS

Findings were relatively homogenous across all three FGDs. Findings are presented according to the two main study objectives and associated themes: *understanding factors underlying premature mortality* amongst MCN groups and *opportunities for interventions to prevent mortality.* Quotes most in agreement are presented in Tables 3 and 4. Quotes from individuals with lived experience of MCN were more likely to focus on their personal experience as opposed to system factors as identified by staff groups. There was a high level of agreement in data collection both within and across the focus groups.

Understanding factors underlying premature mortality in MCN groups

Table 3. Key quotations illustrating subthemes within understanding factors underlying premature mortality in MCN

Theme	Quotes

For peer review only - http://bmjopen.bmj.com/site/about/guidelines.xhtml

Burden of mental ill-health and substance misuse issues	Most of the people I know that's died, their mental health has just been shot to bits, it's all about the drugs. They're taking the drugs because of mental health, is that bad? I'd say more the mental health killed them…the drugs just done that job<br>- Individual with lived experience of MCN
	These [new psychoactive substances] are completely changing the conversation to what they were 10, 15 years ago because these drugs, how they work, how quickly they hit, how quickly they can be produced, how quickly for many of them you're on cloud 9, you're away from it, 15 minutes later you're back as a normal person. Within those 15 minutes what damage you could have done to yourself, to your life, to other people, to other people's lives.<br>- Individual with lived experience of MCN
	And there's a reason why people are taking spice, because oblivion is better than reality. That's the truth of it. It's a much better option facing up to what society is<br>- Individual with lived experience of MCN
	I just find that people who have got mental-health issues and also have addiction problems fall through the gaps, time and time again<br>- Frontline staff
	The waiting list for CAMHS is ridiculous. You've got to be well up there on the scale to get referred. Someone with a little bit of anxiety is not going to get put through to CAMHS, whereas that anxiety will then just carry on getting worse and worse and worse, and then you end up with someone with real mental-health issues<br>- Frontline staff
Lack of hope, stigma, and health-seeking behaviour	You don't see another way…it's just doom and gloom and like you say this one's dead, this one's in prison, there's nothing ever…it's like being in the sort of devil's dungeon, to be honest<br>- Individual with lived experience of MCN
	This is my life, so it's not a care in the world if it is death. Death has got to be better. So to be honest living that life, to some I think death would just be a much easier answer<br>- Individual with lived experience of MCN
	It's a remembrance wall. So all the people in the service that have passed away and every time you go in it's just getting more, you have to squeeze the names in of the people that have been lost and I know them all, I'm going, "Oh so and so has died"<br>- Individual with lived experience of MCN
	Oh you're an addict," the look on their face that it changes visibly, they treat you completely differently, "Oh he knows".<br>- Individual with lived experience of MCN
	I think that word as well, like, junky really boils my blood, heroin addict is much better. Just picking up the junky, junky, junky, that's all we get.<br>- Individual with lived experience of MCN
	… this doctor at the time of [the] appointment isn't going to be able to comprehend even a tiny touch of what your life is.<br>- Individual with lived experience of MCN
	We talked to the young people who have got mental health issues and they're kind of like, "Oh no, I don't want to talk to anyone, I don't want to tell them I've got a problem. I'd rather just smoke some weed or take some grass and I'll be okay.<br>- Manager/Commissioner
Poor navigation and limited	My staff are supposed to spend their time navigating and signposting and supporting people into other services, probably about 60% of their time is now

For peer review only

	spent doing benefits stuff, just so that people have got enough money in their pockets.<br>- Manager/Commissioner
	I'm facing this maze full of doors and every time I open a door, there's another door, sets of doors. There's no coherent structure within the system that says, Here's a person who is asking for help, who's engaging with everything that we're giving, can we please pull this together so we can actually provide the help that this person needs.<br>- Individual with lived experience of MCN
	There are no youth services left…there's nothing left, and that was a huge safety net. It was a learning experience, it was preventive, and it was a place of safety for youth, and it's not there anymore.<br>- Manager/Commissioner
	It's often such a desperate situation that we're having ridiculous conversations that we want someone to be sectioned or we want someone to go to prison just so they're in some kind of contained environment where we feel we can try and manage some of the risks<br>- Manager/Commissioner

Burden of mental ill-health and substance misuse issues

The severe burden of mental ill-health in people with MCN was a recurrent concern and felt to make a substantial contribution to premature mortality, particularly in relation to mental illness alongside substance misuse (dual diagnosis). Participants in the individual with lived experience FGD shared experiences and perceptions of drugs being used as a way for individuals to escape from their day to day circumstances.

Across all three FGDs, there was a perception that people with MCN experienced challenges when trying to access services and support for co-existing mental ill health and substance misuse. Services were set up to deal with either mental health issues, or addiction issues, meaning those with a dual diagnosis failed to receive appropriate support.

Lack of hope, stigma and health-seeking behaviour

Participants with MCN highlighted that experiences of loss were so common that it led to individuals becoming desensitized to deaths and reaching a point of acceptance. Outcomes for individuals experiencing MCN were so often negative this led to a lack of hope that it could ever be any different.

Stigma was discussed within the individual with lived experience FGD in relation to the large role it was perceived to play in health-seeking behaviour. Individuals with lived experience expressed frustration around the facial expressions and stigmatizing nature of the words professionals used to describe different facets of MCN. This fear of stigma along with not being given sufficient time for staff to understand their unique experiences led to individuals avoiding services. FGDs with staff reiterated this perception that many young people they worked with felt intimidated when going to see their General Practitioner and instead chose to self-medicate with illicit substances.

Poor navigation and limited services

For peer review only - http://bmjopen.bmj.com/site/about/guidelines.xhtml

Across all three FGDs, participants expressed problems with navigating service pathways, limited services and having to engage with traditional models of care. Individuals with MCN felt as though they were faced with a series of barriers to gaining appropriate support, which increased the vulnerability of MCN groups to premature mortality. There was a perception that recent reductions in available support services meant that the support was often only provided in extreme circumstances, such as when an individual had reached a crisis point.

Opportunities for interventions to prevent morality in MCN groups

Table 4. Key quotations illustrating themes within understanding factors underlying premature mortality in MCN

Theme	Quotes
Intervention timing	Preventive measures early on may stop the numbers of people coming through with multiple and complex needs. So it's the preventive, it's the community centres, it's the youth centres, it's those things where the learning happens.<br>- Manager/Commissioner
	People leaving prisons, we know the times when you've been drug free and then you transition in and out of prison etc. or transition from being clean for a bit to then lapsing, acute risk there. That would be a flag<br>- Individual with lived experience of MCN
	We all know times of peak vulnerability, they don't need to be necessarily shared emotionally…people leaving prison …loss, bereavement, grief, divorce<br>- Individual with lived experience of MCN
	There's often an inability to exploit windows of opportunity where…support workers will try and get all their ducks in a row. So the mental health stuff, the mental health treatment, housing, benefits, all of that sort of stuff, it's rare that you're going to manage to get all of that sorted in the two hours of window opportunity you've got. Then the ship sails sometimes and you don't know whether that's going to come back again or when it's going to come back again.<br>- Manager/commissioner
Intervention approaches	I think there needs to be focus on it being really a person-centred approach and say, "This isn't working for me at the moment and that's how I would like things to be," and giving them that sense of responsibility<br>- Frontline staff
	There's a connection [as a peer supporter]. Immediately there's a connection but through that connection you feel like you've gotten in and you feel like what you say has a better chance of making a difference to that person<br>- Individual with lived experience of MCN
	I volunteer in services and in statutory services and the differences you see and people's attitudes towards you when they learn that you're an addict or an ex-addict, I'm still an addict. I am an addict, I just don't use. The difference that you see in people's faces when they say, "Oh you're an addict," the look on their face that it changes visibly, they treat you completely differently, "Oh he knows".<br>- Individual with lived experience of MCN
	We exist in a competitive tendering landscape and we need to leave that aside and come together and share good practice and learn from what's happening across the world<br>- Manager/Commissioner

For peer review only - http://bmjopen.bmj.com/site/about/guidelines.xhtml

	We need as people for services to be talking to one another to be sharing our data, to be aware of all of the needs because …that's how we get rounded people by having well rounded service provision.<br>- Individual with lived experience of MCN

Intervention timing

Critical life events (e.g. childhood adversity and bereavement) and transitions (e.g. release from prison) were highlighted as moments of key vulnerability, as well as potential opportunities for intervention. Participants felt that many of the issues encountered by people experiencing MCN were rooted in early childhood. Therefore, improved support for young people who experience adverse childhood experiences could prevent the development and exacerbation of long-term needs. Participants in all focus groups agreed that windows of opportunity (time points of places where services could be provided) are often brief and difficult to take advantage of.

Intervention approaches

Across all FGDs, participants provided suggestions for how interventions could be improved in future. The need for a holistic, person-centred approach was highlighted, acknowledging that a "one-size-fits-all" approach was unlikely to cover the complexity of need or empower individuals to engage. Building a sense of community for people living with MCN was a common suggestion aimed at reducing social exclusion. The value of peer support communities in particular was highlighted among all participants in the FGD with individuals with lived experience of MCN. The need for better collaboration across the multiple agencies involved in supporting people living with MCN was also highlighted. FGDs with staff and managers/commissioners focused more on the financial and competitive element of the current system. In particular, improved communication between services, and the opportunity to share learning across sectors were suggested as ways to reduce missed opportunities for prevention.

DISCUSSION

This is the first study to the best of our knowledge which uses peer research approaches to explore individual and service provider perspectives and experiences on factors that could contribute to high mortality rates for individual living with MCN at a local level. Power within co-produced research can manifest in a number of ways and can result in the focus being placed on capacity building versus reflecting the topic aspirations of peer researchers.(27, 28) In contrast, our study topic was solely determined by peer researchers thereby reflecting their aspirations while providing an opportunity to empower through capacity building as previously suggested. This study used FGDs to gather insights from individuals with lived experience, frontline workers and those responsible for managing and commission services on perspectives into the high mortality rates in MCN populations within the region and opportunities for potential interventions. Across discussions, we found that issues relating to the burden of mental ill-health and substance misuse (dual diagnosis), combined with experiences of stigma and exclusion when accessing services, were perceived as informing premature mortality in this group. Participants highlighted key life events (e.g. childhood adversity) and transitions (e.g. release from prison), as potential opportunities for targeted intervention to better support this population in the future.

For peer review only - http://bmjopen.bmj.com/site/about/guidelines.xhtml

This study provides valuable qualitative insights from both people living with MCN and those delivering and commissioning services on perspectives and experiences on how co-occurring mental ill-health and substance misuse might combine to affect health outcomes and may lead to premature mortality. Fitzpatrick theorises that causes of homelessness occur on at least four levels—economic, housing, interpersonal, and individual—with no one level considered greater than another.(29) The interlocking nature of these causes result in individuals having different experiences of homelessness and some (those experiences MCN) requiring more services and support than others. As others have reported,(18) we found that the reality of many individuals with MCN is a journey dominated by the challenge of navigating a siloed, highly fragmented system, that is ill-equipped to meet their needs. Not unique to this study, are the experiences of many people with MCN finding the co-occurring nature of issues leads to an inability to access services or falling through system "cracks."(19) The experience of facing endless closed doors and no infrastructure in place to provide support irrespective of the access point reiterates the system not being designed for the multi-level adversity MCN populations experience.

Stigma is a well-described phenomenon in this population.(5, 8, 18) Our study highlights that stigma and the associated discrimination experienced by people with MCN also acts as a barrier to accessing support and was perceived as contributing to the high rates or mortality in this population. The perspective on experiences of threat shared by individuals with lived experience, regarding the impact of stigma on health-seeking behaviours and the impact of negative experiences using acute services, may inform subsequent health behaviour and may contribute to decisions leading to premature mortality. If an individual believes they will be dismissed or marginalised by a provider, there is the potential that this may increase the likelihood that they do not pursue services when needed. This notion of group marginalisation may be part of the premature mortality problem, and our qualitative study begins to capture and articulate this.

Experiences across FGDs highlight the struggle within the system to meet current demands and have positive interactions and outcomes for individuals with MCN. This is consistent with current literature.(5) Participants in the focus groups pointed to issues with the present system and identified that early prevention and targeting interventions at "critical life events" (i.e. transitions in service provision or at experiences of heightened adversity) could be important in reducing deaths in MCN groups. Through connecting individuals with support during these 'critical life events' it increases their ability to bounce back and develop resiliency. There was a particular emphasis on creating interventions targeting youth to address concerns earlier on in the prevention spectrum. Evidence highlighting the link between experiences of childhood adversity and later life MCN reiterate the need to create preventative interventions surrounding this time period.(5, 7, 30) Additionally, our findings suggest that future provision should focus on interventions that are developed collaboratively across sectors (5, 31) targeting critical life events (7, 16, 30) using person-centred and trauma-informed practices,(31, 32) and peer support.(33)

The novelty of this study is it has shown the value of listening to and conducting research with individuals with experience of MCN. It enabled an exploration of an issue that directly affects their community and supported understanding of the personal perspective of a handful of people with MCN and their carers on what may have informed premature mortality among their networks, as well as identifying perceived avenues for possible preventive interventions within the local region. This enrichment of the research aligns with existing literature exploring peer

For peer review only - http://bmjopen.bmj.com/site/about/guidelines.xhtml

research use for vulnerable populations or sensitive topics.(8, 20-25) Furthermore, the insights specific to opportunities for service provision take into consideration the lived experience of the target population, which can lead to more equitable service delivery and engagement.(21)

Limitations

As our study was located in the North East of England, our findings may not be generalisable to other regions. The study collected data through three FGDs which included representation from MCN groups and from different sectors of support services. Although the study sample had a broad range of representation, the findings many not be generalizable to other contexts. Nonetheless, the results of this study have provided qualitative insight into perspectives on mortality rates among MCN populations and potential directions for future intervention research.(34) Response bias is a possibility as individuals with stronger views might have been more likely to volunteer participate in a study. Recognising focus groups have their limitations, we provided opportunity for everyone who attended to participate and actively sought input from all participants. We feel that this reduced the potential effects as the group-based data collection allowed for a range of views to be considered and reflected on. There were limitations in the representativeness of individuals with MCN; a recruitment challenge experienced in previous studies.(21) Nonetheless, by engaging peer researchers throughout the research process, we feel this potential limitation was reduced. There is also the risk that participants might have felt more comfortable speaking to an outsider rather than a peer about certain issues.(22) However, as the study focused less on the individual and more on the overarching issues related to mortality rates, this was likely minimal.

CONCLUSIONS

Mortality rates among individuals experiencing MCN in the UK continue to rise due to an interconnected web of disadvantage and exclusion. Findings from this study provide valuable insights from people with lived experience and staff supporting MCN groups on what may be informing premature mortality among this population. Addressing concerns related to dual diagnosis, providing support around critical life events, and investing in early prevention were perceived as ways to mitigate high mortality among individuals experiencing MCN. The intersecting nature of MCN should be factored into the design of future interventions to address the challenges related to service navigation and availability. The need to support existing inequalities related to experiences of MCN and develop effective and sustainable interventions to prevent premature mortality has become even more important in light of the COVID-19 pandemic, which has exacerbated issues around service access, health inequalities, and social isolation.(35)

For peer review only - http://bmjopen.bmj.com/site/about/guidelines.xhtml

Acknowledgements:

We would like to acknowledge the various Peer Researchers from Fulfilling Lives Newcastle Gateshead who contributed to all stages of this study including developing the aim, methods, data collection, and interpretation. As well, we appreciate the time and effort of all participants who offered their views on and experiences of this challenging and emotive topic.

Funding statement:

This work was funded by a small seed grant from Public Health England as part of the Research Hub Initiative. EAA, ELG, GJM, AOD, and SR, are members of Fuse, the Centre for Translational Research in Public Health (www.fuse.ac.uk). Fuse is a UK Clinical Research Collaboration (UKCRC) Public Health Research Centre of Excellence. Funding for Fuse from the British Heart Foundation, Cancer Research UK, National Institute of Health Research, Economic and Social Research Council, Medical Research Council, Health and Social Care Research and Development Office, Northern Ireland, National Institute for Social Care and Health Research (Welsh Assembly Government) and the Wellcome Trust, under the auspices of the UKCRC, is gratefully acknowledged. EAA is supported by the National Institute for Health Research (NIHR) School for Public Health Research (SPHR) Pre-doctoral Fellowship, Grant Reference Number PD-SPH-2015. SR and AOD are members of the National Institute for Health Research (NIHR) Applied Research Collaboration (ARC) North East & North Cumbria Inequality Theme. The views expressed are those of the author(s) and not necessarily those of the NIHR, Department of Health and Social Care, Public Health England, or any of the other funding or organizational bodies.

Declaration of interests:

All authors declare no competing interests.

Data sharing:

Due to the sensitivity of the topic and the numbers of participants, we are not able to share FGD transcripts. Summaries are available from the corresponding author on request.

Author contributions:

Conceptualization: JH, SR, AOD, AB, GJM, ELG and RP
Data acquisition: JH, AB, and SR
Analysis: RP, AB, ELG, GJM, and EAA
Interpretation: All authors contributed to the interpretation of findings.
Writing: All authors contributed to the writing of the paper.
All authors have read and approved the final version to be submitted for consideration for publication.

For peer review only - http://bmjopen.bmj.com/site/about/guidelines.xhtml

References:

1. Marshall L, Finch D, Cairncross L, et al. Mortality and life expectancy trends in the UK: Stalling progress. London: The Health Foundation, 2019. Available: https://reader.health.org.uk/mortality-trends-in-the-uk [Accessed Aug 2020].

2. Marmot M, Allen J, Boyce T, et al. Health equity in England: The marmot review 10 years on. London; 2020. Available: https://www.health.org.uk/publications/reports/the-marmot-review-10-years-on [Accessed Aug 2020].

3. Marmot M. Health equity in England: The marmot review 10 years on. *BMJ* 2020;368:m693. doi: 10.1136/bmj.m693.

4. Office for National Statistics. Drug-related deaths by local authority: England and wales. 2019. Available: www.ons.gov.uk/peoplepopulationandcommunity/birthsdeathsandmarriages/deaths/datasets/drugmisusedeathsbylocalauthority [Accessed Aug 2020].

5. Bramley G, Fitzpatrick S, Edwards J, et al. Hard edges: Mapping severe and multiple disadvantage (England). London: Lankelly Chase Foundation 2015. Available: https://lankellychase.org.uk/wp-content/uploads/2015/07/Hard-Edges-Mapping-SMD-2015.pdf [Accessed Aug 2020].

6. Rosengard A, Laing I, Ridley J, et al. A literature review on multiple and complex needs. Edinburgh: Scottish Executive Social Research 2007. Available: https://www.webarchive.org.uk/wayback/archive/20170112055441/http://www.gov.scot/Publications/2007/01/18133419/15 [Accessed Aug 2020].

7. Fitzpatrick S, Bramley G, Johnsen S. Pathways into multiple exclusion homelessness in seven UK cities. *Urban Stud*2012;50(1):148-68. doi:10.1177/0042098012452329.

8. Terry L, Cardwell V. Refreshing perspectives: Exploring the application of peer research with populations facing severe and multiple disadvantage. London: Revolving Doors Agency & Lankelly Chase 2016. Available: http://www.revolving-doors.org.uk/file/1849/download?token=Yi0tjhmo [Accessed Aug 2020].

9. Fazel S, Khosla V, Doll H, et al. The prevalence of mental disorders among the homeless in western countries: Systematic review and meta-regression analysis. *PLoS Med*2008;5(12):e225. doi:10.1371/journal.pmed.0050225.

10. John A, McGregor J, Jones I, et al. Premature mortality among people with severe mental illness — new evidence from linked primary care data. *Schizophr Res*2018;199:154-62. doi:10.1016/j.schres.2018.04.009.

11. Arendt M, Munk-Jørgensen P, Sher L, et al. Mortality among individuals with cannabis, cocaine, amphetamine, mdma, and opioid use disorders: A nationwide follow-up study of Danish substance users in treatment. *Drug Alcohol Depend*2011;114(2):134-39. doi:10.1016/j.drugalcdep.2010.09.013.

For peer review only - http://bmjopen.bmj.com/site/about/guidelines.xhtml

12. Graham L, Fischbacher CM, Stockton D, et al. Understanding extreme mortality among prisoners: A national cohort study in Scotland using data linkage. *Eur J Public Health*2015;25(5):879-85. doi:10.1093/eurpub/cku25.

13. Thomas B. Homelessness kills: An analysis of the mortality of homeless people in early twenty-first century England. London: Crisis 2012. Available: https://www.crisis.org.uk/media/236798/crisis_homelessness_kills2012.pdf [Accessed Aug 2020].

14. Aldridge RW, Story A, Hwang SW, et al. Morbidity and mortality in homeless individuals, prisoners, sex workers, and individuals with substance use disorders in high-income countries: A systematic review and meta-analysis. *Lancet*2018;391(10117):241-50. doi:10.1016/S0140-6736(17)31869-X.

15. Morrison DS. Homelessness as an independent risk factor for mortality: results from a retrospective cohort study. *Int J Epidemiol*2009;38(3):877-883. doi: http://dx.doi.org/10.1093/ije/dyp160

16. Montgomery AE, Szymkowiak D, Marcus J, et al. Homelessness, unsheltered status, and risk factors for mortality: findings From the 100 000 homes campaign. *Public Health Rep*2016;131(6):765-772. doi: https://doi.org/10.1177/0033354916667501

17. Hjemsæter AJ, Bramness JG, Drake R, et al. Mortality, cause of death and risk factors in patients with alcohol use disorder alone or poly-substance use disorders: A 19-year prospective cohort study. *BMC Psychiatry*2019;19(1):101. doi:10.1186/s12888-019-2077-8.

18. Terry L, Cardwell V. Understanding the whole person. London: Revolving Doors Agency & Lankelly Chase 2015. Available: https://lankellychase.org.uk/wp-content/uploads/2015/12/Understanding-the-whole-person-Part-One.pdf [Accessed Aug 2020].

19. St. Mungo's. Dying on the streets: The case for moving quickly to end rough sleeping. 2018. Available: https://www.mungos.org/app/uploads/2018/06/Dying-on-the-Streets-Report.pdf [Accessed Aug 2020].

20. Woodall J, Cross R, Kinsella K, et al. Using peer research processes to understand strategies to support those with severe, multiple and complex health needs. *Health Education Journal*2019;78(2):176-88. doi:10.1177/0017896918796044.

21. Bonevski B, Randell M, Paul C, et al. Reaching the hard-to-reach: A systematic review of strategies for improving health and medical research with socially disadvantaged groups. *BMC Med Res Methodol*2014;14(1):42-42. doi:10.1186/1471-2288-14-42.

22. Elliott E, Watson AJ, Harries U. Harnessing expertise: Involving peer interviewers in qualitative research with hard-to-reach populations. *Health Expect*2002;5(2):172-78. doi:10.1046/j.1369-6513.2002.00158.x.

For peer review only

23. Brannick T, Coghlan D. In defense of being "native": the case for insider academic research. *Organizational Research Methods*2007;10(1):59-74. doi:10.1177%2F1094428106289253

24. Humprey C. Dilemmas in doing insider research in professional education. *Qualitative social work*2013;12(5);572-586. doi:10.1177%2F1473325012446006

25. Taylor J. The intimate insider: Negotiating the ethics of friendship when doing insider research. *Qualitative research*2011;11(1);3-22. doi:10.1177%2F1468794110384447

26. Braun V, Clarke V. Using thematic analysis in psychology. *Qual Res Psychol*2006;3(2):77-101. doi:10.1191/1478088706qp063oa.

27. Guta A, Flicker S, Roche B. Governing thorugh community allegiance: a qualitative examination of peer research in community-based participatory research. *Crit Public Health*2013;23(4):432-451. doi:10.1080/09581596.2012.761675

28. Foucault M. The subject and power.*Critical inquiry*,1982;8(4):777-795.

29. Fitzpatrick S. Explaining homelessness: a critical realist perspective. *Housing, theory, and society*2005;22(1):1-17. doi:10.1080/14036090510034563

30. McDonagh T. Tackling homelessness and exclusion: Understanding complex lives. York: Joseph Rowntree Foundation 2011. Available: https://www.jrf.org.uk/sites/default/files/jrf/migrated/files/homelessness-exclusion-services-summary.pdf [Accessed Aug 2020].

31. Department for Communities and Local Government. Making every contact count: A joint approach to preventing homelessness. London: DCLG 2012. Available: https://assets.publishing.service.gov.uk/government/uploads/system/uploads/attachment_data/file/7597/2200459.pdf [Accessed Aug 2020].

32. Communities and Local Government. Mental health good practice guide. Meeting the psychological and emotional needs of homeless people. London: CLG and National Mental Health Development Unit 2010. Available:

33. Miler JA, Carver H, Foster R, et al. Provision of peer support at the intersection of homelessness and problem substance use services: A systematic 'state of the art' review. *BMC Public Health*2020;20(1):641. doi:10.1186/s12889-020-8407-4.

34. Boddy CR. Sample size for qualitative research. *Qualitative Market Research: An International Journal*2016;19(4),426-432. doi:10.1108/QMR-06-2016-0053

35. Douglas M, Katikireddi SV, Taulbut M, et al. Mitigating the wider health effects of covid-19 pandemic response. *BMJ*2020;369:m1557. doi:10.1136/bmj.m1557.

For peer review only

Manuscript: Exploring high mortality rates among people with multiple and complex needs: a qualitative study using peer research methods

Consolidated criteria for reporting qualitative studies (COREQ): 32-item checklist

No. Item	Guide questions/description	Reported on Page #
**Domain 1: Research team and reflexivity**		
Personal Characteristics		
1. Interviewer/facilitator	Which author/s conducted the interview or focus group?	4
2. Credentials	What were the researcher's credentials? E.g. PhD, MD	1,4,5,6
3. Occupation	What was their occupation at the time of the study?	1,4,5,6
4. Gender	Was the researcher male or female?	Not reported
5. Experience and training	What experience or training did the researcher have?	4
Relationship with participants		
6. Relationship established	Was a relationship established prior to study commencement?	4
7. Participant knowledge of the interviewer	What did the participants know about the researcher? e.g. personal goals, reasons for doing the research	4
8. Interviewer characteristics	What characteristics were reported about the inter viewer/facilitator? e.g. Bias, assumptions, reasons and interests in the research topic	4,5
**Domain 2: study design**		
Theoretical framework		
9. Methodological orientation and Theory	What methodological orientation was stated to underpin the study? e.g. grounded theory, discourse analysis, ethnography, phenomenology, content analysis	3,10,11
Participant selection		

For peer review only

10. Sampling	How were participants selected? e.g. purposive, convenience, consecutive, snowball	4
11. Method of approach	How were participants approached? e.g. face-to-face, telephone, mail, email	4
12. Sample size	How many participants were in the study?	5
13. Non-participation	How many people refused to participate or dropped out? Reasons?	5
Setting		
14. Setting of data collection	Where was the data collected? e.g. home, clinic, workplace	5
15. Presence of non-participants	Was anyone else present besides the participants and researchers?	5
16. Description of sample	What are the important characteristics of the sample? e.g. demographic data, date	5
Data collection		
17. Interview guide	Were questions, prompts, guides provided by the authors? Was it pilot tested?	5
18. Repeat interviews	Were repeat interviews carried out? If yes, how many?	No
19. Audio/visual recording	Did the research use audio or visual recording to collect the data?	5
20. Field notes	Were field notes made during and/or after the interview or focus group?	5
21. Duration	What was the duration of the interviews or focus group?	5
22. Data saturation	Was data saturation discussed?	5
23. Transcripts returned	Were transcripts returned to participants for comment and/or correction?	5
**Domain 3: analysis and findings**		
Data analysis		
24. Number of data coders	How many data coders coded the data?	6
25. Description of the coding tree	Did authors provide a description of the coding tree?	6
26. Derivation of themes	Were themes identified in advance or derived from the data?	6
27. Software	What software, if applicable, was used to manage the data?	6
28. Participant checking	Did participants provide feedback on the	6

For peer review only

	findings?	
Reporting		
29. Quotations presented	Were participant quotations presented to illustrate the themes/findings? Was each quotation identified? e.g. participant number	7-8, 9-10
30. Data and findings consistent	Was there consistency between the data presented and the findings?	6-10
31. Clarity of major themes	Were major themes clearly presented in the findings?	6-10
32. Clarity of minor themes	Is there a description of diverse cases or discussion of minor themes?	6-10

For peer review only
